# Area deprivation index and segregation on the risk of HIV: a U.S. Veteran case–control study

**DOI:** 10.1016/j.lana.2023.100468

**Published:** 2023-03-21

**Authors:** Abiodun O. Oluyomi, Angela L. Mazul, Yongquan Dong, Donna L. White, Christine M. Hartman, Peter Richardson, Wenyaw Chan, Jose M. Garcia, Jennifer R. Kramer, Elizabeth Chiao

**Affiliations:** a Section of Epidemiology and Population Sciences, Department of Medicine, Baylor College of Medicine, Houston, TX, USA; b Dan L Duncan Comprehensive Cancer Center, Baylor College of Medicine, Houston, TX, USA; c Gulf Coast Center for Precision Environmental Health, Baylor College of Medicine, Houston, TX, USA; d Department of Otolaryngology/Head and Neck Surgery, Washington University School of Medicine, Saint Louis, MO, USA; e VA Health Services Research Center of Innovations in Quality, Effectiveness, and Safety (IQuESt), Michael E. DeBakey VA Medical Center, Houston, TX, USA; f Center for Translational Research in Inflammatory Disease (CTRID), Michael E. DeBakey VA Medical Center, Houston, TX, USA; g Section of Health Services Research, Department of Medicine, Baylor College of Medicine, USA; h Department of Biostatistics and Data Science, School of Public Health, The University of Texas Health Science Center at Houston, USA; i Geriatric Research, Education, and Clinical Center, VA Puget Sound Health Care System and Div. of Geriatrics, Department of Medicine, University of Washington, Seattle, WA, USA; j Department of Epidemiology, Division of Cancer Prevention and Population Sciences, Department of General Oncology, Division of Cancer Medicine, The University of Texas MD Anderson Cancer Center, Houston, TX, USA

**Keywords:** HIV, Racial segregation, United States, Epidemiology

## Abstract

**Background:**

Preventing HIV infection remains a critically important tool in the continuing fight against HIV/AIDS. The primary aim is to evaluate the effect and interactions between a composite area-level social determinants of health measure and an area-level measure of residential segregation on the risk of HIV/AIDS in U.S. Veterans.

**Methods:**

Using the individual-level patient data from the U.S. Department of Veterans Affairs, we constructed a case–control study of veterans living with HIV/AIDS (VLWH) and age-, sex assigned at birth- and index date-matched controls. We geocoded patient's residential address to ascertain their neighborhood and linked their information to two measures of neighborhood-level disadvantage: area deprivation index (ADI) and isolation index (ISOL). We used logistic regression to estimate the odds ratio (OR) and 95% confidence interval (CI) for comparing VLWH with matched controls. We performed analyses for the entire U.S. and separately for each U.S. Census division.

**Findings:**

Overall, living in minority-segregated neighborhoods was associated with a higher risk of HIV (OR: 1.88 (95% CI: 1.79–1.97) while living in higher ADI neighborhoods was associated with a lower risk of HIV (OR: 0.88; 95% CI: 0.84–0.92). The association between living in a higher ADI neighborhood and HIV was inconsistent across divisions, while living in minority-segregated neighborhoods was consistently associated with increased risk across all divisions. In the interaction model, individuals from low ADI and high ISOL neighborhoods had a higher risk of HIV in three divisions: East South Central; West South Central, and Pacific.

**Interpretation:**

Our results suggest that residential segregation may prevent people in disadvantaged neighborhoods from protecting themselves from HIV independent from access to health care. There is the need to advance knowledge about the neighborhood-level social-structural factors that influence HIV vulnerability toward developing interventions needed to achieve the goal of ending the HIV epidemic.

**Funding:**

10.13039/100000054US National Cancer Institute.

**Research in context**

**Evidence before this study**

Given the increasing disparities in HIV risk and mortality between White and Black Americans, preventing HIV infection remains a critical tool in the continuing fight against HIV/AIDS. Meanwhile, current evidence suggests that examining geographic neighborhood-level factors provides a critical framework for disseminating and implementing HIV prevention. We identified English language papers, published within the last decade, that studied associations between living in socioeconomically disadvantaged neighborhoods and higher risk of HIV diagnosis. We highlight several below: a cross-sectional study with a national representative sample collected between 2006 and 2010 reported that core-based statistical area-level racial residential segregation was associated with risky sexual behaviour among Non-Hispanic Blacks. A longitudinal study of heterosexual Black adults and adolescents in US metropolitan statistical areas (MSAs) found that racial residential segregation was associated with HIV infection. We also reviewed previous work on the relative significance of neighborhood sociodemographic and racial composition. In one study, even when accounting for differences in socioeconomic status, authors found that Black men living in areas highly concentrated with other Black people had higher rates of delayed HIV diagnoses than those in less concentrated areas. This study also found that neighborhoods with the highest (relative to lowest) Black racial concentration had higher relative risk of late HIV diagnosis among men and women independent of socioeconomic deprivation. To summarise, a recent (2022) systematic review that examined the relationship between neighborhood-level factors and HIV vulnerability synthesized 55 relevant articles published between 2007 and 2017. They spotlighted consistent findings of associations between neighborhood disadvantage and increased risk of HIV. However, most of these studies did not evaluate the effects of access to health care, and few studies adjusted for other social determinants of health factors.

**Added value of this study**

In this analysis, we assessed the relationships and interactions between a measure of neighborhood-level socioeconomic deprivation (ADI), neighborhood residential segregation (isolation index), and the risk of HIV in U.S. Veterans, the largest single-payer, integrated healthcare system in the U.S. We found that living in minority-segregated neighborhoods increased the risk of HIV in all U.S. geographic divisions, even after adjusting for racial identity and ADI. Conversely, the association between living in a higher ADI neighborhood and HIV was inconsistent. We used a large national dataset of VA healthcare users which allowed us to adjust for regional trends, guaranteed reliable HIV diagnosis data, and minimised the potential confounding of healthcare access that is often challenging to measure in U.S. population studies. Unlike other previous studies that relied on aggregated surveillance HIV diagnosis, we used individual-level HIV diagnosis data from a medical record system that allowed us to conduct a case–control study. We also included relevant individual-level factors in our statistical modelling.

**Implications of all the available evidence**

In the U.S., the V.A. system is considered similar to a universal healthcare system, where access to health is theoretically equitable. While equal access to health care should theoretically translate to better health outcomes, other social determinants of health (SDOH) factors have also been shown to have great impacts on health outcomes. Our analysis shows that using data from a population with equal access to health care and despite adjusting for general measures of SDOH (i.e., ADI), residential segregation predicted clear differences for HIV risk in all U.S. regions. Our study supports other population-based and community-based studies evaluating the impact of systemic racism and residential segregation on HIV incidence. Altogether, these studies emphasise the need to advance knowledge about the neighborhood-level social-structural factors that influence HIV vulnerability, and to develop targeted HIV prevention interventions needed to achieve the goal of ending the HIV epidemic.

## Introduction

Although the introduction of antiretroviral therapy (ART) and pre-exposure prophylaxis have mitigated HIV/AIDS-related mortality, persons living with HIV/AIDS need continuous access to appropriate medical care. Social injustice and unequal access to care have driven HIV risk and mortality disparities. Indeed, disparities in mortality rates between White and Black Americans increased recently than in the pre-ART era, with decreases in HIV-related mortality among whites nearly twice that of Black Americans.^1^ Consequently, preventing HIV infection remains a critical tool in the continuing fight against HIV/AIDS. Current evidence suggests that people of color in the U.S. who live in socioeconomically disadvantaged neighborhoods are at higher risk for HIV and have higher AIDS-related mortality.^2^^,^^3^

The COVID19 pandemic has highlighted systemic healthcare disparities and the high burden of infectious disease among people of color in the United States.^4^ Social determinants of health (SDOH) are the conditions in which people are born, grow, work, live, and age that affect health. These forces and structures include economic policies, development agendas, social norms, racism, climate change, and political systems. Examining geographic neighborhood-level factors provides a critical framework for disseminating and implementing HIV prevention. Residential neighborhood-level socioenvironmental factors, including poverty, low educational attainment, substandard housing, and lack of employment opportunities,^5^^,^^6^ affect health. Several studies have observed HIV-associated racial health disparities such as lower access and adherence to treatment,^7^, ^8^, ^9^, ^10^ quality of care upon hospitalisation,^7^ and quality of life post-diagnosis (survivorship),^10^ and mortality.,^7^^,^^11^ housing segregation has recently been associated with adverse health outcomes.^12^, ^13^, ^14^ While the significance of the SDoH in the U.S. healthcare discourse has been growing,^15^, ^16^, ^17^ the specific impact of housing segregation and other SDoH on HIV risk at the national level remains poorly understood.^18^

The national U.S. Veterans Health Administration (VHA) is the largest integrated healthcare system in the U.S. Military veterans eligible for health care benefits through the VHA receive integrated services with equal access to care. This analysis aims to evaluate the effect and interactions between a composite area-level SDoH measure and an area-level measure of residential segregation among and the risk of HIV/AIDS in U.S. Veterans.

## Methods

### Study setting

We constructed a frequency matched case–control study from a previously described HIV cancer incidence cohort study.^19^ This cohort was constructed with individual-level patient data from the U.S. Department of Veterans Affairs (V.A.) Corporate Data Warehouse (CDW). The CDW includes yearly enrollment information, laboratory test results, pharmacy, and inpatient and outpatient utilization as indicated by procedure (CPT codes) and diagnosis codes (ICD-9 codes) for all V.A. users nationwide. Enrollment data containing comprehensive and time-updating address information was used to locate patients inside their U.S. census tracts. The Institutional Review Board of Baylor College of Medicine and the Michael E. DeBakey VA Medical Center approved this study.

#### Case definition

We identified cases with a HIV diagnosis from October 1, 1999, and December 31, 2016. Kramer et al. detailed how the HIV diagnosis was defined.^19^ Briefly, to be considered a veteran living with HIV/AIDS (VLWH), patients must be aged ≥18 years old and fulfilled two of three HIV-specific criteria: lab tests, including HIV antibody with ELISA or Western blot, HIV viral load, and CD4 count; prescription data for HIV therapy; and presence of inpatient or outpatient codes for HIV. These criteria yield a high sensitivity (95.2%) and positive predictive value (93.5%).^19^ We used the earliest date of the HIV diagnosis as the study index date.

#### Control definition and sampling

We sampled controls from more than 10 million unique patients in the VA CDW databases during the study period. Potential HIV-negative veteran participants required at least two outpatient appointments in the CDW database between October 1, 1999, and December 31, 2016, without a positive HIV lab test, an ART prescription, or an ICD code for HIV. We matched HIV-negative patients with VLWH based on sex assigned at birth, age at the index date within two years, and an outpatient visit during the same month as the index date for the VLWH. Then, we used a randomised selection algorithm to select four frequency-based matches for each HIV-infected patient (N = 46,788 VLWH and N = 170,911 HIV-negative veterans).

#### Geocoding cases and controls

**Fig.1:**
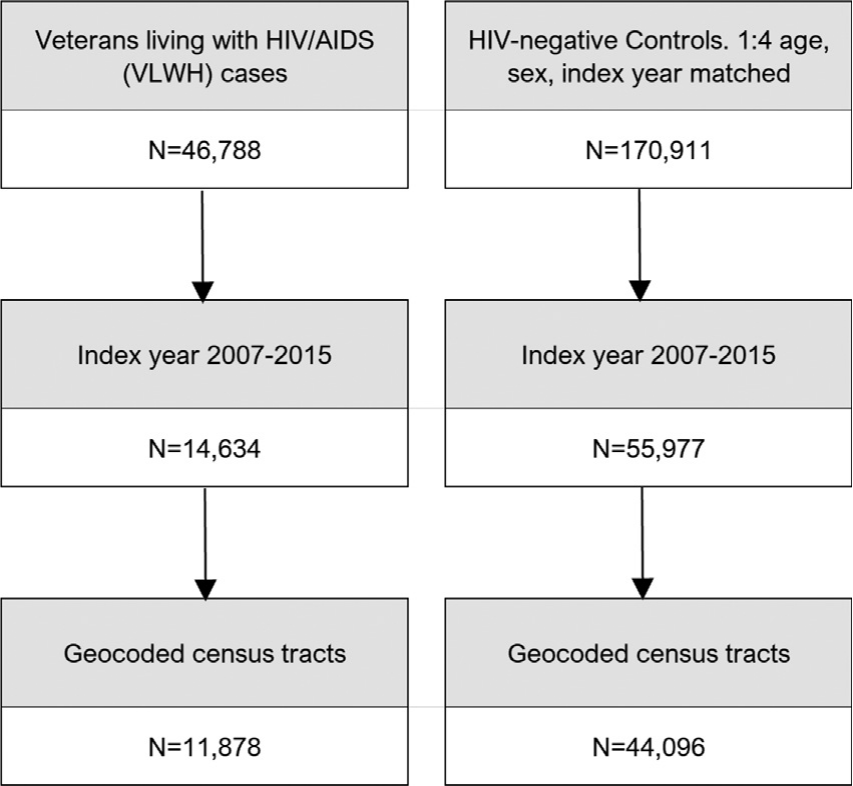
Selection of study population from the U.S. Department of Veterans Affairs (V.A.) Corporate Data Warehouse.

We restricted index dates from January 1, 2007, to December 31, 2015 (Fig. 1) and found 14,634 VLWH, of which 11,878 had a census tract FIPS – an 11-digit number that uniquely identifies each census tract. Among the controls, 55,977 HIV-negative veterans had an index date from 2007 to 2015, and 44,096 had a census tract FIPS. Participants were missing census tracts due to incomplete geocoding from missing or incomplete residential addresses or a residential address outside of the contiguous United States.

### Explanatory variables

We used ArcGIS Pro (Esri Corporation, Redlands, California) to operationalize and ascertain spatially referenced neighborhood characteristics.^20^ Our primary explanatory variables were the neighborhood socioeconomic disadvantage and residential segregation indexes, computed at the census tract level. With an optimum population of 4000 residents or 1600 housing units, the census tract is a small and relatively permanent statistical subdivision designed to be homogeneous.^21^ We employed the U.S. Census American Community Survey (ACS) 5-year estimate data to compute these indexes.^22^

#### Socioeconomic disadvantage

The Area Deprivation Index (ADI), developed and validated by Singh,^23^ is a composite measure of neighborhood socioeconomic disadvantage,^24^ that encompasses poverty, housing, employment, and education.^23^ In short, we used previously defined weights and coefficients for 17 socioeconomic indicators drawn from these four major categories from the ACS 5-year estimate to calculate ADI.^25^ The ADI was calculated separately for each ACS 5-year estimate, starting with the 2005–2009 5-year estimate and ending on the 2011–2015. The ADI was computed for any ACS episode assigned to the end year of that episode. For example, the ADI for patients with a 2015 index year was computed for a census tract based on the 2011–2015 ACS episode. There were two exceptions. First, patients whose index years were 2007 or 2008 were assigned the ADI based on the inaugural 5-year 2005–2009 ACS. Second, between 2009 and 2015, if the ADI was missing for the ACS episode that matched a patient index year, the next available ADI closest to that ACS episode was used. Overall, 95.6% of patients were matched to the expected ACS year (eTable 1.1; Supplement).

#### Residential segregation

The isolation index (ISOL) measures exposure, the degree of potential contact between groups within a specific geographic area. ISOL assesses the degree of potential contact within a neighborhood between Non-Hispanic (N.H.) Whites and any minority sub-group combined. The 0-to-1 ISOL range expresses the probability that minority sub-group individuals share the same geographic unit. We followed the formula presented in a U.S. Census official report on residential segregation for the current proposal.^26^ We used race frequencies from census block groups as measured by indexing the percentage of neighborhood co-residents who are also minority group members. The ISOL was calculated separately for four ACS episodes. The ISOL scores used the 2005–2009 ACS were assigned to patients whose index years were 2007, 2008, and 2009. The three remaining ACS episodes and their corresponding index years were: 2007–2011 ACS (2010 and 2011 index years), 2009–2013 ACS (2012 and 2013 index years), and 2011–2015 ACS (2014 and 2015 index years). If the ISOL was missing for the ACS episode that matched a patient index year, the next available ISOL closest to that ACS episode was used. Overall, 97.6% of patients were matched to the expected ACS episode (eTable 1.2; Supplement).

**Fig. 2:**
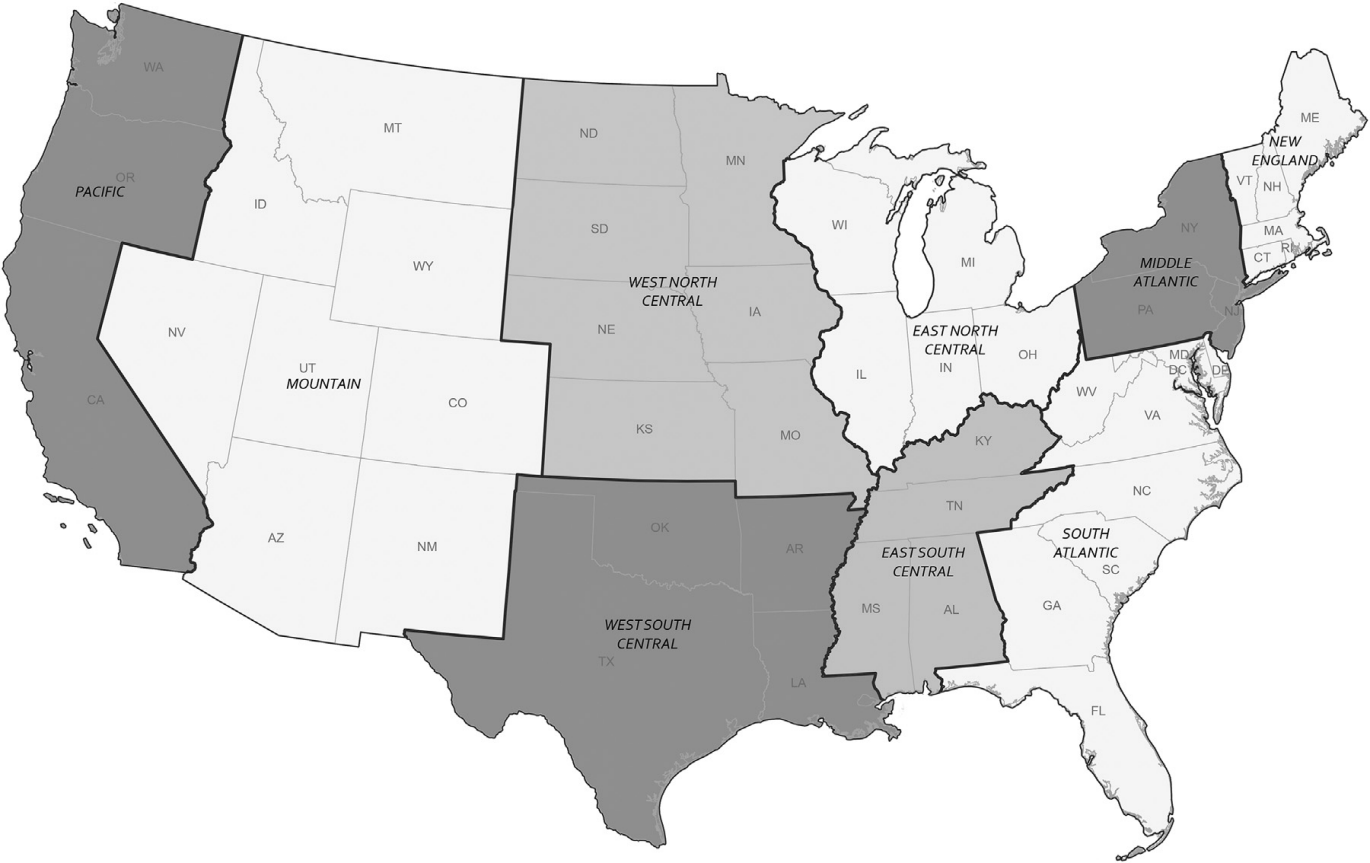
States contained inside the U.S. Census Bureau-designated divisions (N = 9 divisions). Only states inside the continental U.S. were used for our analysis. **Notes:** U.S. Census Bureau-designated divisions are grouped under four regions—described below: Region 1: NORTHEAST. **Division 1: New England** (Connecticut, Maine, Massachusetts, New Hampshire, Rhode Island, Vermont), **Division 2: Middle Atlantic** (New Jersey, New York, Pennsylvania). Region 2: MIDWEST. **Division 3: East North Central** (Indiana, Illinois, Michigan, Ohio, Wisconsin), **Division 4: West North Central** (Iowa, Kansas, Minnesota, Missouri, Nebraska, North Dakota, South Dakota). Region 3: SOUTH. **Division 5: South Atlantic** (Delaware, District of Columbia, Florida, Georgia, Maryland, North Carolina, South Carolina, Virginia, West Virginia), **Division 6: East South Central** (Alabama, Kentucky, Mississippi, Tennessee), **Division 7: West South Central** (Arkansas, Louisiana, Oklahoma, Texas). Region 4: PACIFIC. **Division 8: Mountain** (Arizona, Colorado, Idaho, New Mexico, Montana, Utah, Nevada, Wyoming), and **Division 9: Pacific** (Alaska, California, Hawaii, Oregon, Washington). Alaska and Hawaii not included in the current analysis.

We arranged the patients’ census tracts into their respective census divisions to assess geographic differences across the U.S. The Census Bureau has designated the grouping of states into the nine census divisions since 1910 (Fig. 2).^27^^,^^28^ For analysis, the ADI and ISOL raw scores were transformed into division-specific quintile classifications. The quintile classification was later dichotomized as low (Q1-Q3) vs. high (Q4-Q5) ADI. High ADI scores represent greater neighborhood disadvantage (i.e., low socioeconomic status), while high ISOL scores represent higher residential segregation.

#### Individual-level covariates

We selected specific individual-level characteristics in the VA CDW data, including age, sex, race/ethnicity (NH White, NH Black, Hispanic, other, unknown), and homelessness (yes, no). These covariates were *a priori* selected due to their relationship with HIV and ADI. The Census Bureau-designated divisions (Fig. 2) were covariates in the overall national analysis.

### Data analysis

We used logistic regression to estimate the adjusted odds ratios (OR) and 95% confidence interval (CI) for comparing VLWH with matched HIV-negative veterans with ADI and ISOL as the primary exposure while adjusting for frequency-matched sex, age and index year, and other covariates including race/ethnicity, homeless, division, and residence in a metropolitan versus non-metropolitan census tract. We also performed the same analyses stratified by each division. Finally, we examined the interaction between ADI and the isolation index. We used neighborhoods with low ADI (i.e, high SES) and low isolation index (i.e, low segregation) as the reference category for the interaction term analysis. We also calculated the estimated effect of ISOL INDX within levels of ADI. All statistical analyses used SAS software version 9.2.

### Role of the funding source

This study was funded and supported by grant R01CA206476 (Drs Chiao and Kramer; P.I.s) from the 10.13039/100000054National Cancer Institute. Grant CIN13-413 from the Houston Veterans Affairs Health Services Research and Development Center of Innovations. Grant P30 CA125123 from the 10.13039/100007856Baylor College of Medicine 10.13039/100008527Dan L. Duncan Cancer Center. Grant K01MD013897 (Dr. Mazul) from the National Institute on Minority Health and Health Disparities. Dr. Oluyomi's effort was supported in part by grant P30ES030285 (Dr. Walker; P.I.) from the National Institute of Environmental Health. The funding source had no role in the design and conduct of the study; collection, management, analysis, and interpretation of the data; preparation, review, or approval of the manuscript; and decision to submit the manuscript for publication.

## Results

Our study consisted of 11,878 VLWH cases and 44,096 HIV-negative veteran controls (Table 1; Fig. 1). Our study population was mostly non-Hispanic White [N = 33,436 (59.73%)], male [N = 53,724 (95.98%)], and lived in a metropolitan census tract [N = 42,031 (75.09%)]. VLWH were far more likely to live in minority-segregated neighborhoods [N = 8213 (69.14%)] compared to HIV-negative veterans [N = 17,606 (39.93%)]. VLWH were also more likely to be Black, homeless, and live in the South Atlantic and metro census tracts than controls. Detailed characteristics of the study population for the entire U.S. are shown in eTable 2, and by division in eTable 3. Of note, the Middle Atlantic had the most VLWH [N = 1004 (79.62%)] living in minority-segregated neighborhoods, while the Pacific division had the least [N = 945 (53.78%)]. East South Central was the only division where most VLWH lived in high ADI [N = 408 (54.33%)] census tracts. There were variations by race. Over half the VLWH were Black in the Middle Atlantic, South Atlantic, and East South Central.

**Table 1:** Characteristics of study patients by HIV status and association between HIV incidence and measures of socioeconomic deprivation (ADI) and residential segregation (Isolation Index).

Characteristics	Overall (N = 55,974)	HIV + cases (N = 11,878)	HIV- controls (N = 44,096)	aOR^a^ (95%CI)	P
ADI					
Q1 - Q3	33,396 (59.66)	6932 (58.36)	26,464 (60.01)	Ref	
Q4 - Q5	22,578 (40.34)	4649 (41.64)	17,632 (39.99)	0.88 (0.84–0.92)	<0.001
ISOL INDX					
Q1 - Q3	30,025 (53.64)	3617 (30.45)	26,408 (59.89)	Ref	
Q4 - Q5	25,819 (46.13)	8213 (69.14)	17,606 (39.93)	1.88 (1.79–1.97)	<0.001
Race/ethnicity					
NH White	33,436 (59.73)	4397 (37.02)	29,039 (65.85)	Ref	
NH Black	13,987 (24.99)	6116 (51.49)	7871 (17.85)	3.44 (3.26–3.63)	<0.001
Hispanic	3245 (5.80)	739 (6.22)	2506 (5.68)	1.31 (1.19–1.44)	<0.001
Other/unknown	5306 (9.48)	2512 (5.27)	4680 (10.61)	0.72 (0.66–0.79)	<0.001
Homeless					
No	53,730 (95.99)	10,525 (88.61)	43,205 (97.98)	Ref	
Yes	2244 (4.01)	1353 (11.39)	891 (2.02)	4.21 (3.83–4.63)	<0.001
Rural/urban					
Metropolitan	42,031 (75.09)	10,039 (84.52)	31,992 (72.55)	Ref	
Not Metropolitan	13,943 (24.91)	1839 (15.48)	12,104 (27.45)	0.71 (0.66–0.75)	<0.001
Division					
Pacific	7395 (13.21)	1757 (14.79)	5638 (12.79)	Ref	
New England	1859 (3.32)	242 (2.04)	1617 (3.67)	0.50 (0.43–0.59)	<0.001
Mid Atlantic	4769 (8.52)	1261 (10.62)	3508 (7.96)	0.86 (0.78–0.94)	0.001
East North Central	7323 (13.08)	1038 (8.74)	6285 (14.25)	0.49 (0.44–0.53)	<0.001
West North Central	3585 (6.40)	358 (3.01)	3227 (7.32)	0.41 (0.36–0.47)	<0.001
South Atlantic	15,318 (27.37)	4193 (35.30)	11,125 (25.23)	0.88 (0.82–0.95)	<0.001
East South Central	3979 (7.11)	751 (6.32)	3228 (7.32)	0.60 (0.54–0.67)	<0.001
West South Central	7241 (12.99)	1655 (13.93)	5616 (12.74)	0.82 (0.76–0.90)	<0.001
Mountain	4475 (7.99)	623 (5.24)	3852 (8.74)	0.61 (0.55–0.68)	<0.001

aOR: Adjusted Odds Ratio; P: p-value; ADI: Area Deprivation Index; ISOL INDX: Isolation Index.

National sample of U.S. Veterans from 2007 to 2015.

a Mutually adjusted odds ratio. Age, sex and index year are not included since they are matching factors.

### Overall national analysis of the association between ADI, isolation index and risk of HIV

First, the entire study population was entered into a single model to estimate the risk of HIV across the U.S. (Table 1). In an adjusted model, living in higher ADI neighborhoods was associated with a lower risk of HIV (OR: 0.88; 95% CI: 0.84–0.92) and living in minority-segregated neighborhoods was associated with a higher risk of HIV (OR: 1.88 (95% CI: 1.79–1.97). Furthermore, living in non-metropolitan neighborhoods was inversely associated with HIV [OR: 0.71 (95% CI: 0.66–0.75)]. We also found that Black [OR: 3.44 (95% CI: 3.26–3.63)] and Hispanic [OR: 1.31 (95% CI: 1.19–1.44)] race/ethnicity was associated with an increased risk of HIV.

### Division-specific analysis of the association between ADI, isolation index and risk of HIV

**Fig. 3:**
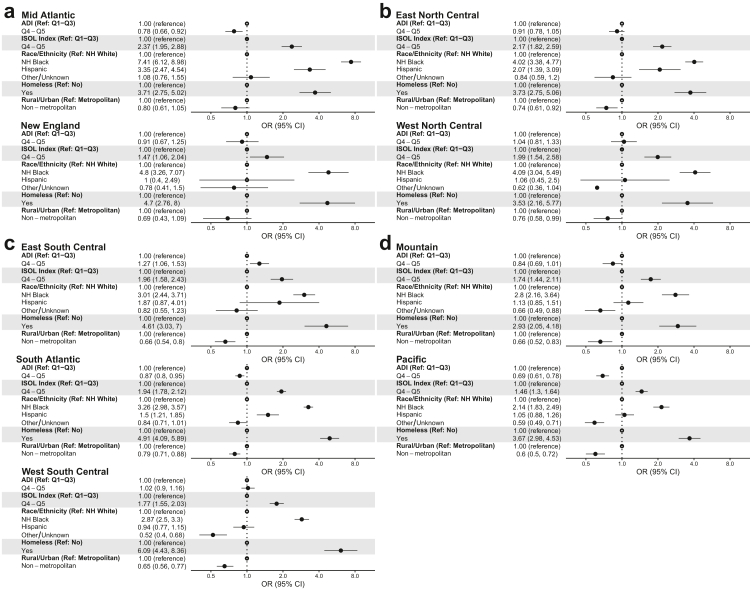
Division-specific association between HIV incidence and measures of socioeconomic deprivation (ADI) and residential segregation (Isolation Index). National sample of U.S. Veterans from 2007-2015. U.S. Census regions: (**a**) = Northeast, (**b**) = Midwest, (**c**) = South, (**d**) = West.

In each U.S. Census division, living in minority-segregated neighborhoods was associated with an increased risk of HIV. However, the association between ADI and the risk of HIV was variable (Fig. 3). In the Mid-Atlantic, South Atlantic, and Pacific divisions, living in high ADI neighborhoods was associated with a lower risk of HIV. Only in the East South Central division was higher ADI associated with HIV. Hispanic ethnicity was associated with increased risk in Mid Atlantic, East North Central, South Atlantic. Black race and homelessness were associated with an increased risk of HIV across all divisions. In a sensitivity analysis, we compared the full model that included both ADI and isolation index with a smaller model that had only the ADI; without isolation index. In this case, the ADI had positive relationship with HIV in two divisions as opposed to one in the full model and negative relationship in two divisions as opposed to three in the full model. Otherwise, when adjusted for isolation index, ADI had no significant association in five divisions (eTable 4 for national models and eTable 5 for division-specific models).

### Assessment of interaction between ADI and isolation index

Three of the nine census divisions studied showed significant interactions between ADI and the isolation index (Table 2; eTable 6). When compared with the reference category low ADI (i.e, high SES) and low isolation index (i.e, low segregation), individuals from low ADI and high isolation index neighborhoods had a higher risk of HIV in all the three divisions [OR: 1.63 (95% CI: 1.27–2.11) in East South Central; OR: 1.45 (95% CI: 1.23–1.71) in West South Central; and OR: 1.20 (95% CI: 1.05–1.39) in Pacific]. The high ADI and low segregation neighborhoods had a lower risk of HIV in West South Central (OR: 0.71; 95% CI: 0.57–0.88) and Pacific (OR: 0.51; 95% CI: 0.42–0.60) while the high ADI and high segregation neighborhoods had a higher risk of HIV in East South Central (OR: 2.44; 95% CI: 1.90–3.13) and West South Central (OR: 1.83; 95% CI: 1.57–2.14). Within strata of ADI, high segregation was associated with an increased risk of HIV. The estimated effect of segregation was stronger among Veterans living in low SES (i.e., high ADI) neighborhoods.

**Table 2:** Multivariable Interaction term analysis between Area Deprivation Index (ADI) and Isolation Index (ISOL INDX) and HIV Incidence in U.S. Regions that demonstrated a significant ADI and ISOL INDX interaction.

		East South Central		West South Central		Pacific	
		aOR (95%CI)^a^	Stratum Specific aOR (95%CI)^a^	aOR (95%CI)^a^	Stratum Specific aOR (95%CI)^a^	aOR (95%CI)^a^	Stratum Specific aOR (95%CI)^a^
ADI	ISOL INDX						
Q1 - Q3 (High SES)	Q1 - Q3 (Low Segregation)	Ref	Ref	Ref	Ref	Ref	Ref
	Q4 - Q5 (High Segregation)	1.63 (1.27–2.11)	1.63 (1.27–2.11)	1.45 (1.23–1.71)	1.45 (1.23–1.71)	1.20 (1.05–1.39)	1.20 (1.05–1.39)
Q4 - Q5 (Low SES)	Q1 - Q3 (Low Segregation)	0.88 (0.63–1.25)	Ref	0.71 (0.57–0.88)	Ref	0.51 (0.42–0.60)	Ref
	Q4 - Q5 (High Segregation)	2.44 (1.90–3.13)	2.77 (1.95–3.92)	1.83 (1.57–2.14)	2.60 (2.07–3.26)	1.12 (0.95–1.32)	2.21 (1.80–2.71)

aOR: Adjusted Odds Ratio; P: p-value; ADI: Area Deprivation Index; ISOL INDX: Isolation Index.

a Adjusted odds ratio: race/ethnicity, homeless, age, sex, rural/urban, and index year are not included since they are matching variables.

## Discussion

Our study joins recent and growing research examining various aspects of the relationships between neighborhood-level demographic and socioeconomic factors and HIV. In this case–control analysis, we assessed the relationships between a measure of neighborhood-level socioeconomic deprivation (ADI), neighborhood residential segregation (isolation index), and the risk of HIV in U.S. Veterans who use the nationwide V.A. healthcare system. The ADI has been used to examine disease risk factors,^29^ predict healthcare utilization,^30^ and understand healthcare disparities.^23^ The isolation index is also associated with various health outcomes, including all-cause and cancer mortality.^31^, ^32^, ^33^, ^34^ We found that living in minority-segregated neighborhoods increased the risk of HIV in all U.S. geographic divisions, even after adjusting for race and ADI. Conversely, the association between living in a higher ADI neighborhood and HIV was inconsistent.

In the U.S., the V.A. system is generally considered similar to a universal healthcare system, where access to health is theoretically equitable. While equal access to health care should theoretically translate to better health outcomes, other social determinants of health (SDOH) factors have also been shown to have great (if not greater) impacts on health outcomes. Our analysis shows that using data from a population with equal access to health care and in spite of adjusting for general measures of SDOH (as measured by the ADI), residential segregation predicted clear and distinct differences across the U.S. for HIV risk. Differences in minority-segregated neighborhoods in every division, but the effect estimates varied considerably from the Pacific division's 1.46 (95% CI: 1.30–1.64) to the Mid-Atlantic division's 2.37 (95% CI: 1.95–2.88). Our findings reinforce specific and significant geographic differences in how neighborhood-level factors act as the risk factor for HIV. Previous work and anecdotal commentary have highlighted economic condition as a significant risk factor for HIV incidence, with a consensus that lower economic standing is associated with higher HIV risk.^18^ Our data demonstrate that neighborhood residential segregation outperformed the ADI as a significant risk of HIV in this population even after accounting for neighborhood socioeconomic status and race/ethnicity and the fact that the Veterans in this study had uniform access to care through the VA. These findings demonstrate that racial/ethnic segregation in U.S. neighborhoods influences HIV risks through yet unmeasured mechanisms.

Our study suggests that segregation is more critical than socioeconomic status, and supports other population-based studies evaluating the impact of systemic racism and residential segregation on HIV incidence. Ibragmimov et al.^35^ utilised data from 95 large U.S. metropolitan statistical areas (MSAs) from 2008 to 2015 to show that racial residential segregation was associated with HIV infection among heterosexual Black adults and adolescents. In particular, a one standard deviation decrease in baseline isolation was associated with a 16.2% reduction in the rate of new HIV diagnoses. In contrast, one standard deviation reduction in isolation over time was associated with a 4.6% decrease in the rate of new HIV diagnoses. Although this study evaluated the mediation from certain other SDOH variables, including education, employment, and poverty, this population-level analysis did not account for access to medical care. Another population-based study utilizing the New York City HIV registry found that adjusting for differences in socioeconomic status, Black men living in areas highly concentrated with other Black people had higher rates of delayed HIV diagnoses than those in less concentrated areas.^36^ This study also found that neighborhoods with the highest (relative to lowest) Black racial concentration had a higher relative risk of late HIV diagnosis among men and women independent of income inequality and socioeconomic deprivation, as well as prevalence and accessibility of HIV testing.^36^ Hispanic immigrants – who tend to live in segregated neighborhoods – account for a third of all HIV diagnoses among Latinos and are at greater risk than their U.S.-born peers for delayed diagnosis and presentation to care.^37^, ^38^, ^39^, ^40^

Deliberate and explicit racism through discriminatory housing practices created segregated neighborhoods with concentrated poverty, crime, and limited upward mobility.^41^ These communities often have limited access to health-promoting resources, such as educational opportunities, medical care, and chronic stress due to financial strain and over-policing. The “hyper-incarceration of Black men” has been hypothesized to increase HIV risk in majority-Black neighborhoods by causing imbalanced sex ratios and partner (un)availability for heterosexual Black women might shift the power structure to favor available men when negotiating sexual partnerships in the community.^42^, ^43^, ^44^ Maintaining and establishing monogamous committed relationships with incarcerated men may be difficult given polities that restrict access to safe and affordable housing based on criminal and legal involvement.^45^ In addition, although racial segregation increased the risk of HIV in all geographic regions, the attenuation of this effect in different U.S. census regions suggest that specific state-based health-related, or other social policies may play a mitigating role.

Our study has significant strengths. We used a large national dataset to examine regional differences across the U.S. Using VA healthcare users guaranteed reliable HIV diagnosis data while minimizing the potential confounding of healthcare access that is often challenging to measure in U.S. population studies. Our use of census tract to represent the neighborhood is noteworthy. Representing neighborhoods with larger geographic units (e.g., zip code or CBSA) may obscure the heterogeneity of the studied neighborhood factors. Small geographic units (e.g., census tracts) provide more accurate estimates of neighborhood-level characteristics.^46^, ^47^, ^48^ While our study builds upon similar studies that used larger geographic units, including MSAs,^35^ cities,^49^ and zip codes,^50^ these studies relied on aggregated surveillance HIV diagnosis. In comparison, we had individual-level HIV diagnosis data from a medical record system that allowed us to conduct a case–control study. Furthermore, we included relevant individual-level factors in our statistical modelling.

Limitations include the potential for exposure-outcome misclassification despite our efforts to time-match the neighborhood-level factors and HIV diagnoses. Longitudinal assessment of the ADI and isolation index was impossible without the historical residential address data. Also, although our findings may not be generalised to the entire U.S. population, our observations may be more pronounced in the general population with less equitable access to healthcare. Finally, despite our robust approach, we cannot rule out other unexamined factors, anddraw any causal links for our findings.

### Conclusion

The health disparity among Americans of color is not due to individual choices but results from structural and systemic racism embedded in American society. The historical legacy of discriminatory housing practices (i.e., redlining) and structural racism affects virtually every aspect of where people live in the U.S. Our results suggest that structural racism and residential segregation impact HIV risk for people of color independent of access to health care and the potential effect of living in disadvantaged and impoverished neighborhoods. Further work is needed to understand the etiologies of increased HIV risk and poor outcomes due to racial segregation to develop interventions that address neighbourhood and structural context to reduce HIV risk.

## Contributors

Abiodin Oluyomi, Yongquan Dong and Peter Richardson verified the data and had access to raw data.

Drs Oluyomi, Mazul, Kramer, Chiao had full access to all of the data in the study and take responsibility for the integrity of the data and the accuracy of the data analysis. Drs Oluyomi and Mazul contributed equally and share first authorship.

Concept and design: Oluyomi, Mazul, Chiao, Kramer

Acquisition, analysis, or interpretation of data: Oluyomi, Mazul, Dong, White, Hartman, Richardson, Chan, Garcia, Kramer, Chiao

Drafting of the manuscript: Oluyomi, Mazul, Dong, Kramer, Chiao

Critical revision of the manuscript for important intellectual content: All authors

Statistical analysis: Mazul, Dong, Hartman, Richardson

Obtained funding: Oluyomi, Kramer, Chiao

Administrative, technical, or material support: Oluyomi, Mazul, Dong, White, Richardson, Chan, Garcia, Kramer, Chiao

Supervision: Kramer, Chiao

Abiodin Oluyomi, Angela Mazul and Elizabeth Chiao were primarily responsible for the decision to submit, but all authors accepted responsibility for the final decision to submit for publication.

## Data sharing statement

A Limited Dataset (LDS) can be created and shared pursuant to a Data Use Agreement (DUA) that: 1) indicates adherence to any applicable Informed Consent provisions, 2) appropriately limits use of the dataset and 3) prohibits the recipient from taking steps to allow for identifying or re-identifying any individual whose data are included in the dataset. NOTE: An LDS does not necessarily imply de-identified data per HIPAA.

## Editor note

The Lancet Group takes a neutral position with respect to territorial claims in published maps and institutional affiliations.

## Declaration of interests

None reported.
