# Supplementary material for: Area deprivation index and segregation on the risk of HIV: a U.S. Veteran case–control study
Source: Lancet Reg Health Am. 2023 Mar 21;20:100468. doi: 10.1016/j.lana.2023.100468 (PMC10041556; doi:10.1016/j.lana.2023.100468)
Supplement: Supplementary material [file mmc1.docx]

**Supplementary Materials: Online-only material**

**Title:** Residential Segregation is associated with HIV/AIDS in US Veterans, A National Study.

- **eTable 1.1:** Breakdown of how patients were matched with American Community Survey (ACS) episodes for the the Area Deprivation Index (ADI).
- **eTable 1.2:** Breakdown of how patients were matched with American Community Survey (ACS) episodes for the Isolation Index.
- **eTable 2:** Characteristics of study patients by HIV status; all patients. National sample of US Veterans from 2007-2015.
- **eTable 3:** Characteristics of study patients by census regions and HIV status. National sample of US Veterans from 1999 – 2015.
- **eTable 4:** Sensitivity analysis that compared the full model (Model 1: ADI and isolation index) with a smaller model (Model 2: ADI only, without isolation index). Overall national analysis.
- **eTable 5:** Sensitivity analysis that compared the full model (Model 1: ADI and isolation index) with a smaller model (Model 2: ADI only, without isolation index). Division-specifc analysis.
- **eFigure1:** Division-specific association between HIV incidence and measures of socioeconomic deprivation (ADI) and residential segregation (Isolation Index). National sample of US Veterans from 2007-2015.

**eTable 1.1:** Breakdown of how patients were matched with American Community Survey (ACS) episodes for the Area Deprivation Index (ADI).

| Index Year | No. Patients. | Index ACS Episode | | | -1 ACS Episode | | | +1 ACS Episode | | | Missing | |
| --- | --- | --- | --- | --- | --- | --- | --- | --- | --- | --- | --- | --- |
|  |  | ACS Episode Used | No. Patients. Matched | % Total Matched | ACS Episode Used | No. Patients. Matched | % Total Matched | ACS Episode Used | No. Patients. Matched | % Total Matched | No. Patients. | Percent |
| 2007, 2008 2009 | 19266 | 2005-2009 | 19232 | 99.82% | N/A |  |  | 2006-2010 | 34 | 0.18% | 0 | 0% |
| 2010 | 7035 | 2006-2010 | 7010 | 99.64% | 2005-2009 | 5 | 0.07% | 2007-2011 | 20 | 0.28% | 0 | 0% |
| 2011 | 6814 | 2007-2011 | 6792 | 99.68% | 2006-2010 | 3 | 0.04% | 2008-2012 | 19 | 0.28% | 0 | 0% |
| 2012 | 7370 | 2008-2012 | 6235 | 84.60% | 2007-2011 | 7 | 0.09% | 2009-2013 | 1128 | 15.31% | 0 | 0% |
| 2013 | 6947 | 2009-2013 | 5999 | 86.35% | 2008-2012 | 4 | 0.06% | 2010-2014 | 944 | 13.59% | 0 | 0% |
| 2014 | 6291 | 2010-2014 | 6269 | 99.65% | 2009-2013 | 6 | 0.10% | 2011-2015 | 16 | 0.25% | 0 | 0% |
| 2015 | 2251 | 2011-2015 | 2235 | 99.29% | 2010-2014 | 16 | 0.71% | N/A |  |  | 0 | 0% |

**eTable 1.2:** Breakdown of how patients were matched with American Community Survey (ACS) episodes for the Isolation Index.

| Index Years   (Range) | No. Patients. | Index ACS Episode | | | -1 ACS Episode | | | +2 ACS Episode | | | +4 ACS Episode | | | Missing | |
| --- | --- | --- | --- | --- | --- | --- | --- | --- | --- | --- | --- | --- | --- | --- | --- |
|  |  | ACS Episode Used | No. Patients. Matched | % Total Matched | ACS Episode Used | No. Patients. Matched | % Total Matched | ACS Episode Used | No. Patients. Matched | % Total Matched | ACS Episode Used | No. Patients. Matched | % Total Matched | No. Patients. | Percent |
| 2007, 2008, 2009 | 19266 | 2005-2009 | 19046 | 98.86% | N/A |  |  | 2010-2011 | 201 | 1.04% | 2012-2013 | 8 | 0.04% | 11 | 0.06% |
| 2010, 2011 | 13849 | 2007-2011 | 13745 | 99.25% | 2007-2009 | 0 | 0.00% | 2012-2013 | 64 | 0.46% | 2014-2015 | 6 | 0.04% | 34 | 0.25% |
| 2012, 2013 | 14317 | 2009-2013 | 13311 | 92.97% | 2010-2011 | 0 | 0.00% | 2014-2015 | 964 | 6.73% | N/A |  |  | 42 | 0.29% |
| 2014, 2015 | 8542 | 2011-2015 | 8466 | 99.11% | 2012-2013 | 23 | 0.27% | N/A |  |  | N/A |  |  | 53 | 0.62% |

**eTable 2:** Characteristics of study patients by HIV status; all patients. National sample of US Veterans from 2007-2015.

|  | Overall (N=55974) | HIV+ (N=11878) | HIV- (N=44096) |
| --- | --- | --- | --- |
| ADI (n(%)) |  |  |  |
| Q1 - Q3 | 33396 (59.66) | 6932 (58.36) | 26464 (60.01) |
| Q4 - Q5 | 22578 (40.34) | 4649 (41.64) | 17632 (39.99) |
| ISOL INDX *(n(%))* |  |  |  |
| Q1 - Q3 | 30025 (53.64) | 3617 (30.45) | 26408 (59.89) |
| Q4 - Q5 | 25819 (46.13) | 8213 (69.14) | 17606 (39.93) |
| Missing | 130 (0.23) | 48 (0.40) | 82 (0.19) |
| Race/Ethnicity *(n(%))* |  |  |  |
| NH White | 33436 (59.73) | 4397 (37.02) | 29039 (65.85) |
| NH Black | 13987 (24.99) | 6116 (51.49) | 7871 (17.85) |
| Hispanic | 3245 (5.80) | 739 (6.22) | 2506 (5.68) |
| Other/Unknown | 5306 (9.48) | 2512 (5.27) | 4680 (10.61) |
| Age (n(%)) |  |  |  |
| 18-34 | 9655 (17.25) | 2165 (18.23) | 7490 (16.99) |
| 35-44 | 9786 (17.48) | 2173 (18.29) | 7613 (17.26) |
| 45-54 | 17612 (31.47) | 3784 (31.86) | 13828 (31.36) |
| 55-64 | 14642 (26.16) | 2898 (24.40) | 11744 (26.63) |
| 65+ | 4277 (7.64) | 858 (7.22) | 3419 (7.75) |
| Sex (n(%)) |  |  |  |
| Female | 2250 (4.02) | 406 (3.42) | 1844 (4.18) |
| Male | 53724 (95.98) | 11472 (96.58) | 42252 (95.82) |
| Homeless *(n(%))* |  |  |  |
| No | 53730 (95.99) | 10525 (88.61) | 43205 (97.98) |
| Yes | 2244 (4.01) | 1353 (11.39) | 891 (2.02) |
| Rural/Urban *(n(%))* |  |  |  |
| Metropolitan | 42031 (75.09) | 10039 (84.52) | 31992 (72.55) |
| Not Metropolitan | 13943 (24.91) | 1839 (15.48) | 12104 (27.45) |
| Division |  |  |  |
| Pacific | 7395 (13.21) | 1757 (14.79) | 5638 (12.79) |
| New England | 1859 (3.32) | 242 (2.04) | 1617 (3.67) |
| Mid Atlantic | 4769 (8.52) | 1261 (10.62) | 3508 (7.96) |
| East North Central | 7323 (13.08) | 1038 (8.74) | 6285 (14.25) |
| West North Central | 3585 (6.40) | 358 (3.01) | 3227 (7.32) |
| South Atlantic | 15318 (27.37) | 4193 (35.30) | 11125 (25.23) |
| East South Central | 3979 (7.11) | 751 (6.32) | 3228 (7.32) |
| West South Central | 7241 (12.99) | 1655 (13.93) | 5616 (12.74) |
| Mountain | 4475 (7.99) | 623 (5.24) | 3852 (8.74) |

**eTable 3:** Characteristics of study patients by census regions and HIV status. National sample of US Veterans from 1999 – 2015.

|  | New England | |  | Middle Atlantic | |  | East North Central | |
| --- | --- | --- | --- | --- | --- | --- | --- | --- |
|  | HIV+ (N=242) | HIV- (N=1617) |  | HIV+ (N=1261) | HIV- (N=3508) |  | HIV+ (N=1038) | HIV- (N=6285) |
| ADI (n(%)) |  |  |  |  |  |  |  |  |
| Q1 - Q3 | 146 (60.33) | 970 (59.99) |  | 861 (68.28) | 2105 (60.01) |  | 561 (54.05) | 3770 (59.98) |
| Q4 - Q5 | 96 (39.67) | 647 (40.01) |  | 400 (31.72) | 1403 (39.99) |  | 477 (45.95) | 2515 (40.02) |
| ISOL INDX *(n(%))* |  |  |  |  |  |  |  |  |
| Q1 – Q3 | 95 (39.26) | 968 (59.86) |  | 222 (17.61) | 2092 (59.64) |  | 268 (25.82) | 3767 (59.94) |
| Q4 – Q5 | 147 (60.74) | 645 (39.89) |  | 1004 (79.62) | 1394 (39.74) |  | 769 (74.08) | 2511 (39.95) |
| Missing | 0 | 4 (0.25) |  | 35 (2.78) | 22 (0.63) |  | 1 (0.10) | 7 (0.11) |
| Race/Ethnicity *(n(%))* |  |  |  |  |  |  |  |  |
| NH White | 152 (62.81) | 1350 (83.49) |  | 306 (24.27) | 2556 (72.86) |  | 457 (44.03) | 4934 (78.50) |
| NH Black | 73 (30.17) | 101 (6.25) |  | 809 (64.16) | 528 (15.05) |  | 508 (48.94) | 809 (12.87) |
| Hispanic | 6 (2.48) | 46 (2.84) |  | 100 (7.93) | 160 (4.56) |  | 35 (3.37) | 130 (2.07) |
| Other/Unknown | 11 (4.55) | 120 (7.42) |  | 46 (3.65) | 264 (7.53) |  | 38 (3.66) | 412 (6.56) |
| Age (n(%)) |  |  |  |  |  |  |  |  |
| 18-34 | 31 (12.81) | 277 (17.13) |  | 163 (12.93) | 630 (17.96) |  | 166 (15.99) | 968 (15.40) |
| 35-44 | 38 (15.70) | 226 (13.98) |  | 172 (13.64) | 487 (13.88) |  | 194 (18.69) | 962 (15.31) |
| 45-54 | 79 (32.64) | 469 (29.00) |  | 437 (34.66) | 1040 (29.65) |  | 338 (32.56) | 1867 (29.71) |
| 55-64 | 61(25.21) | 471 (29.13) |  | 360 (28.55) | 1007 (28.71) |  | 259 (24.95) | 1879 (29.90) |
| 65+ | 33 (13.64) | 174 (10.76) |  | 129 (10.23) | 344 (9.81) |  | 81 (7.80) | 609 (9.69) |
| Sex (n(%)) |  |  |  |  |  |  |  |  |
| Female | 8 (3.31) | 49 (3.03) |  | 44 (3.49) | 105 (2.99) |  | 23 (2.22) | 194 (3.09) |
| Male | 234 (96.69) | 1568 (96.97) |  | 1217 (96.51) | 3403 (97.01) |  | 1015 (97.78) | 6091 (96.91) |
| Homeless *(n(%))* |  |  |  |  |  |  |  |  |
| No | 208 (85.95) | 1576 (97.46) |  | 1072 (85.01) | 3414 (97.32) |  | 922 (88.82) | 6183 (98.38) |
| Yes | 34 (14.05) | 41 (2.54) |  | 189 (14.99) | 94 (2.68) |  | 116 (11.18) | 102 (1.62) |
| Rural/Urban *(n(%))* |  |  |  |  |  |  |  |  |
| Metropolitan | 212 (87.60) | 1241 (76.75) |  | 1166 (92.47) | 2824 (80.50) |  | 895 (86.22) | 4430 (70.49) |
| Not Metropolitan | 30 (12.40) | 376 (23.25) |  | 95 (7.53) | 684 (19.50) |  | 143 (13.78) | 1855 (29.51) |

**eTable 3 (contd.):** Characteristics of study patients by census regions and HIV status. National sample of US Veterans from 1999 – 2015.

|  | South Atlantic | |  | East South Central | |  | West South Central | |
| --- | --- | --- | --- | --- | --- | --- | --- | --- |
|  | HIV+ (N=4193) | HIV- (N=11125) |  | HIV+ (N=751) | HIV- (N=3228) |  | HIV+ (N=1655) | HIV- (N=5616) |
| ADI (n(%)) |  |  |  |  |  |  |  |  |
| Q1 - Q3 | 2314 (55.19) | 6678 (60.03) |  | 343 (45.67) | 1937 (60.01) |  | 903 (54.56) | 3371 (60.02) |
| Q4 - Q5 | 1879 (44.81) | 4447 (39.97) |  | 408 (54.33) | 1291 (39.99) |  | 752 (45.44) | 2245 (39.98) |
| ISOL INDX *(n(%))* |  |  |  |  |  |  |  |  |
| Q1 - Q3 | 1366 (32.58) | 6658 (59.85) |  | 204 (27.16) | 1934 (59.91) |  | 614 (37.10) | 3368 (59.97) |
| Q4 – Q5 | 2823 (67.33) | 4441 (39.92) |  | 546 (72.70) | 1289 (39.93) |  | 1039 (62.78) | 2245 (39.98) |
| Missing | 4 (0.10) | 26 (0.23) |  | 1 (0.13) | 5 (0.15) |  | 2 (0.12) | 3 (0.05) |
| Race/Ethnicity *(n(%))* |  |  |  |  |  |  |  |  |
| NH White | 1135 (27.07) | 6219 (55.90) |  | 239 (31.82) | 2080 (64.44) |  | 594 (35.89) | 3106 (55.31) |
| NH Black | 2750 (65.59) | 3453 (31.04) |  | 472 (62.85) | 814 (25.22) |  | 816 (49.31) | 1119 (19.93) |
| Hispanic | 133 (3.17) | 400 (3.60) |  | 9 (1.20) | 35 (1.08) |  | 167 (10.09) | 700 (12.46) |
| Other/Unknown | 175 (4.17) | 1053 (9.47) |  | 31 (4.13) | 299 (9.26) |  | 78 (4.71) | 691 (12.30) |
| Age (n(%)) |  |  |  |  |  |  |  |  |
| 18-34 | 715 (17.05) | 1663 (14.95) |  | 134 (17.84) | 465 (14.41) |  | 385 (23.26) | 1123 (20.00) |
| 35-44 | 792 (18.89) | 2076 (18.66) |  | 148 (19.71) | 623 (19.30) |  | 352 (21.27) | 1162 (20.69) |
| 45-54 | 1428 (34.06) | 3894 (35.00) |  | 247 (32.89) | 1065 (32.99) |  | 458 (27.67) | 1689 (30.07) |
| 55-64 | 1010 (24.09) | 2739 (24.62) |  | 182 (24.23) | 849 (26.30) |  | 357 (21.57) | 1319 (23.49) |
| 65+ | 248 (5.91) | 751 (6.76) |  | 40 (5.33) | 226 (7.00) |  | 103 (6.22) | 323 (5.75) |
| Sex (n(%)) |  |  |  |  |  |  |  |  |
| Female | 200 (4.77) | 622 (5.59) |  | 35 (4.66) | 126 (3.90) |  | 51 (3.08) | 274 (4.88) |
| Male | 3993 (95.23) | 10503 (94.41) |  | 716 (95.34) | 3102 (96.10) |  | 1604 (96.92) | 5342 (95.12) |
| Homeless *(n(%))* |  |  |  |  |  |  |  |  |
| No | 3729 (88.93) | 10925 (98.20) |  | 680 (90.55) | 3186 (98.70) |  | 1510 (91.24) | 5551 (98.84) |
| Yes | 464 (11.07) | 200 (1.80) |  | 71 (9.45) | 42 (1.30) |  | 145 (8.76) | 65 (1.16) |
| Rural/Urban *(n(%))* |  |  |  |  |  |  |  |  |
| Metropolitan | 3467 (82.69) | 8326 (74.84) |  | 556 (74.03) | 1970 (61.03) |  | 1403 (84.77) | 4115 (73.27) |
| Not Metropolitan | 726 (17.31) | 2799 (25.16) |  | 195 (25.97) | 1258 (38.97) |  | 252 (15.23) | 1501 (26.73) |

**eTable 3 (contd.):** Characteristics of study patients by census regions and HIV status. National sample of US Veterans from 1999 – 2015.

|  | West North Central | |  | Mountain | |  | Pacific | |
| --- | --- | --- | --- | --- | --- | --- | --- | --- |
|  | HIV+ (N=358) | HIV- (N=3227) |  | HIV+ (N=623) | HIV- (N=3852) |  | HIV+ (N=1757) | HIV- (N=5638) |
| ADI (n(%)) |  |  |  |  |  |  |  |  |
| Q1 - Q3 | 195 (54.47) | 1936 (59.99) |  | 370 (59.39) | 2310 (59.97) |  | 1221 (69.49) | 3383 (60.00) |
| Q4 - Q5 | 163 (45.53) | 1291 (40.01) |  | 253 (40.61) | 1542 (40.03) |  | 536 (30.51) | 2255 (40.00) |
| ISOL INDX *(n(%))* |  |  |  |  |  |  |  |  |
| Q1 - Q3 | 118 (32.96) | 1935 (59.96) |  | 271 (43.50) | 2308 (59.92) |  | 807 (45.93) | 3378 (59.91) |
| Q4 - Q5 | 240 (67.04) | 1290 (39.98) |  | 352 (56.50) | 1539 (39.95) |  | 945 (53.78) | 2252 (39.94) |
| Missing |  | 2 (0.06) |  |  | 5 (0.13) |  | 5 (0.28) | 8 (0.14) |
| Race/Ethnicity *(n(%))* |  |  |  |  |  |  |  |  |
| NH White | 224 (62.57) | 2657 (82.34) |  | 379 (60.83) | 2672 (69.37) |  | 911 (51.85) | 3465 (61.46) |
| NH Black | 112 (31.28) | 216 (6.69) |  | 114 (18.30) | 230 (5.97) |  | 462 (26.29) | 601 (10.66) |
| Hispanic | 6 (1.68) | 58 (1.80) |  | 69 (11.08) | 366 (9.50) |  | 214 (12.18) | 611 (10.84) |
| Other/Unknown | 16 (4.47) | 296 (9.17) |  | 61 (9.79) | 584 (15.16) |  | 170 (9.68) | 961 (17.05) |
| Age (n(%)) |  |  |  |  |  |  |  |  |
| 18-34 | 62 (17.32) | 518 (16.05) |  | 128 (20.55) | 710 (18.43) |  | 381 (21.68) | 1136 (20.15) |
| 35-44 | 62 (17.32) | 515 (15.96) |  | 125 (20.06) | 687 (17.83) |  | 290 (16.51) | 875 (15.52) |
| 45-54 | 113 (31.56) | 934 (28.94) |  | 186 (29.86) | 1179 (30.61) |  | 498 (28.34) | 1691 (29.99) |
| 55-64 | 87 (24.30) | 958 (29.69) |  | 144 (23.11) | 1021 (26.51) |  | 438 (24.93) | 1501 (26.62) |
| 65+ | 34 (9.50) | 302 (9.36) |  | 40 (6.42) | 255 (6.62) |  | 150 (8.54) | 435 (7.72) |
| Sex (n(%)) |  |  |  |  |  |  |  |  |
| Female | 6 (1.68) | 104 (3.22) |  | 13 (2.09) | 151 (3.92) |  | 26 (1.48) | 219 (3.88) |
| Male | 352 (98.32) | 3123 (96.78) |  | 610 (97.91) | 3701 (96.08) |  | 1731 (98.52) | 5419 (96.12) |
| Homeless *(n(%))* |  |  |  |  |  |  |  |  |
| No | 323 (90.22) | 3175 (98.39) |  | 568 (91.17) | 3748 (97.30) |  | 1513 (86.11) | 5447 (96.61) |
| Yes | 35 (9.78) | 52 (1.61) |  | 55 (8.83) | 104 (2.70) |  | 244 (13.89) | 191 (3.39) |
| Rural/Urban *(n(%))* |  |  |  |  |  |  |  |  |
| Metropolitan | 259 (72.35) | 1826 (56.59) |  | 502 (80.58) | 2667 (69.24) |  | 1579 (89.87) | 4593 (81.47) |
| Not Metropolitan | 99 (27.65) | 1401 (43.41) |  | 121 (19.42) | 1185 (30.76) |  | 178 (10.13) | 1045 (18.53) |

**eTable 4:** Sensitivity analysis that compared the full model (Model 1: ADI and isolation index) with a smaller model (Model 2: ADI only, without isolation index). Overall national analysis.

|  | **Model 1** |  |  | **Model2** |  |
| --- | --- | --- | --- | --- | --- |
| National | OR (95%CI) | P |  | OR (95%CI) | P |
| ADI |  |  |  |  |  |
| Q1 - Q3 | Ref |  |  | Ref |  |
| Q4 - Q5 | 0.88 (0.84 – 0.92) | <0.001 |  | 0.98 (0.93 – 1.02) | <0.322 |
| ISOL INDX |  |  |  |  |  |
| Q1 - Q3 | Ref |  |  |  |  |
| Q4 - Q5 | 1.88 (1.79 – 1.97) | <0.001 |  |  |  |
| Race/Ethnicity |  |  |  |  |  |
| NH White | Ref |  |  | Ref |  |
| NH Black | 3.44 (3.26 – 3.63) | <0.001 |  | 4.43 (4.21 – 4.66) | <0.001 |
| Hispanic | 1.31 (1.19 – 1.44) | <0.001 |  | 1.63 (1.49 – 1.79) | <0.001 |
| Other/Unknown | 0.72 (0.66 – 0.79) | <0.001 |  | 0.81 (0.74 – 0.89) | <0.001 |
| Age |  |  |  |  |  |
| 18-34 | Ref |  |  | Ref |  |
| 35-44 | 0.97 (0.90 – 1.05) | 0.427 |  | 0.95 (0.88 – 1.02) | 0.175 |
| 45-54 | 0.83 (0.78 – 0.89) | <0.001 |  | 0.83 (0.78 – 0.88) | <0.001 |
| 55-64 | 0.93 (0.87 – 0.99) | 0.040 |  | 0.92 (0.86 – 0.98) | 0.014 |
| 65+ | 1.16 (1.05 – 1.28) | 0.003 |  | 1.14 (1.04 – 1.25) | 0.007 |
| Sex |  |  |  |  |  |
| Female | Ref |  |  | Ref |  |
| Male | 1.56 (1.38 – 1.75) | <0.001 |  | 1.56 (1.39 – 1.76) | <0.001 |
| Homeless |  |  |  |  |  |
| No | Ref |  |  | Ref |  |
| Yes | 4.21 (3.83 – 4.63) | <0.001 |  | 4.36 (3.96 – 4.79) | <0.001 |
| Rural/Urban |  |  |  |  |  |
| Metropolitan | Ref |  |  | Ref |  |
| Not Metropolitan | 0.71 (0.66 – 0.75) | <0.001 |  | 0.64 (0.60 – 0.68) | <0.001 |
| Division |  |  |  |  |  |
| Pacific | Ref |  |  | Ref |  |
| New England | 0.50 (0.43 – 0.59) | <0.001 |  | 0.54 (0.46 – 0.62) | <0.001 |
| Mid Atlantic | 0.86 (0.78 – 0.94) | 0.001 |  | 0.92 (0.84 – 1.01) | 0.081 |
| East North Central | 0.49 (0.44 – 0.53) | <0.001 |  | 0.52 (0.47 – 0.57) | <0.001 |
| West North Central | 0.41 (0.36 – 0.47) | <0.001 |  | 0.45 (0.39 – 0.51) | <0.001 |
| South Atlantic | 0.88 (0.82 – 0.95) | <0.001 |  | 0.87 (0.81 – 0.93) | <0.001 |
| East South Central | 0.60 (0.54 – 0.67) | <0.001 |  | 0.62 (0.56 – 0.69) | <0.001 |
| West South Central | 0.82 (0.76 – 0.90) | <0.001 |  | 0.82 (0.75 – 0.89) | <0.001 |
| Mountain | 0.61 (0.55 – 0.68) | <0.001 |  | 0.63 (0.56 – 0.70) | <0.001 |
| Index Year |  |  |  |  |  |
| 2007 – 2009 | Ref |  |  | Ref |  |
| 2010 – 2012 | 1.02 (0.96 – 1.07) | 0.589 |  | 1.00 (0.95 – 1.06) | 0.874 |
| 2013 – 2015 | 1.09 (1.03 – 1.16) | 0.004 |  | 1.09 (1.03 – 1.15) | 0.004 |

**eTable 5:** Sensitivity analysis that compared the full model (Model 1: ADI and isolation index) with a smaller model (Model 2: ADI only, without isolation index). Division-specifc analysis.

|  | **Model1** |  |  | **Model2** |  |
| --- | --- | --- | --- | --- | --- |
| New England | OR (95%CI) | P |  | OR (95%CI) | P |
| ADI |  |  |  |  |  |
| Q1 - Q3 | Ref |  |  | Ref |  |
| Q4 - Q5 | 0.91 (0.67 – 1.25) | 0.559 |  | 0.96 (0.70 – 1.31) | 0.794 |
| ISOL INDX |  |  |  |  |  |
| Q1 - Q3 | Ref |  |  |  |  |
| Q4 - Q5 | 1.47 (1.06 – 2.04) | 0.022 |  |  |  |
| Race/Ethnicity |  |  |  |  |  |
| NH White | Ref |  |  | Ref |  |
| NH Black | 4.80 (3.26 – 7.07) | <0.001 |  | 5.50 (3.80 – 7.96) | <0.001 |
| Hispanic | 1.00 (0.40 – 2.49) | 0.998 |  | 1.12 (0.46 – 2.75) | 0.804 |
| Other/Unknown | 0.78 (0.41 – 1.50) | 0.453 |  | 0.77 (0.40 – 1.48) | 0.435 |
| Age |  |  |  |  |  |
| 18-34 | Ref |  |  | Ref |  |
| 35-44 | 1.62 (0.94 – 2.79) | 0.081 |  | 1.56 (0.91 – 2.68) | 0.107 |
| 45-54 | 1.40 (0.87 – 2.24) | 0.168 |  | 1.35 (0.84 – 2.17) | 0.210 |
| 55-64 | 1.41 (0.87 – 2.29) | 0.168 |  | 1.35 (0.83 – 2.19) | 0.229 |
| 65+ | 2.20 (1.26 – 3.85) | 0.006 |  | 2.10 (1.20 – 3.66) | 0.009 |
| Sex |  |  |  |  |  |
| Female | Ref |  |  | Ref |  |
| Male | 1.27 (0.54 – 2.99) | 0.589 |  | 1.30 (0.56 – 3.05) | 0.542 |
| Homeless |  |  |  |  |  |
| No | Ref |  |  | Ref |  |
| Yes | 4.70 (2.76 – 8.00) | <0.001 |  | 4.91 (2.88 – 8.36) | <0.001 |
| Rural/Urban |  |  |  |  |  |
| Metropolitan | Ref |  |  | Ref |  |
| Not Metropolitan | 0.69 (0.43 – 1.09) | 0.111 |  | 0.59 (0.38 – 0.91) | 0.017 |
| Index Year |  |  |  |  |  |
| 2007 – 2009 | Ref |  |  | Ref |  |
| 2010 – 2012 | 1.16 (0.82 – 1.63) | 0.404 |  | 1.14 (0.81 – 1.60) | 0.461 |
| 2013 – 2015 | 0.91 (0.62 – 1.33) | 0.613 |  | 0.91 (0.62 – 1.34) | 0.638 |
|  |  |  |  |  |  |
| Mid Atlantic | OR (95%CI) | P |  | OR (95%CI) | P |
| ADI |  |  |  |  |  |
| Q1 - Q3 | Ref |  |  | Ref |  |
| Q4 - Q5 | 0.78 (0.66 – 0.92) | 0.003 |  | 0.77 (0.65 – 0.91) | 0.002 |
| ISOL INDX |  |  |  |  |  |
| Q1 - Q3 | Ref |  |  |  |  |
| Q4 - Q5 | 2.37 (1.95 – 2.88) | <0.001 |  |  |  |
| Race/Ethnicity |  |  |  |  |  |
| NH White | Ref |  |  | Ref |  |
| NH Black | 7.41 (6.12 – 8.98) | <0.001 |  | 11.31 (9.55 – 13.40) | <0.001 |
| Hispanic | 3.35 (2.47 – 4.54) | <0.001 |  | 4.83 (3.62 – 6.43) | <0.001 |
| Other/Unknown | 1.08 (0.76 – 1.55) | 0.664 |  | 1.39 (0.99 – 1.96) | 0.057 |
| Age |  |  |  |  |  |
| 18-34 | Ref |  |  | Ref |  |
| 35-44 | 1.13 (0.85 – 1.52) | 0.399 |  | 1.11 (0.83 – 1.47) | 0.489 |
| 45-54 | 1.12 (0.87 – 1.43) | 0.377 |  | 1.13 (0.88 – 1.43) | 0.335 |
| 55-64 | 1.28 (1.00 – 1.64) | 0.052 |  | 1.27 (1.00 – 1.63) | 0.054 |
| 65+ | 1.76 (1.28 – 2.41) | <0.001 |  | 1.82 (1.34 – 2.48) | 0.001 |
| Sex |  |  |  |  |  |
| Female | Ref |  |  | Ref |  |
| Male | 1.03 (0.68 – 1.58) | 0.886 |  | 1.02 (0.68 – 1.55) | 0.912 |
| Homeless |  |  |  |  |  |
| No | Ref |  |  | Ref |  |
| Yes | 3.71 (2.75 – 5.02) | 0.001 |  | 3.74 (2.77 – 5.05) | <0.001 |
| Rural/Urban |  |  |  |  |  |
| Metropolitan | Ref |  |  | Ref |  |
| Not Metropolitan | 0.80 (0.61 – 1.05) | 0.101 |  | 0.68 (0.52 – 0.88) | 0.003 |
| Index Year |  |  |  |  |  |
| 2007 – 2009 | Ref |  |  | Ref |  |
| 2010 – 2012 | 0.91 (0.76 – 1.10) | 0.345 |  | 0.93 (0.77 – 1.12) | 0.445 |
| 2013 – 2015 | 1.01 (0.83 – 1.23) | 0.930 |  | 1.06 (0.87 – 1.28) | 0.575 |
|  |  |  |  |  |  |
| East North Central | OR (95%CI) | P |  | OR (95%CI) | P |
| ADI |  |  |  |  |  |
| Q1 - Q3 | Ref |  |  | Ref |  |
| Q4 - Q5 | 0.91 (0.78 – 1.05) | 0.191 |  | 1.01 (0.87 – 1.17) | 0.919 |
| ISOL INDX |  |  |  |  |  |
| Q1 - Q3 | Ref |  |  |  |  |
| Q4 - Q5 | 2.17 (1.82 – 2.59) | <0.001 |  |  |  |
| Race/Ethnicity |  |  |  |  |  |
| NH White | Ref |  |  | Ref |  |
| NH Black | 4.02 (3.38 – 4.77) | <0.001 |  | 5.57 (4.75 – 6.53) | <0.001 |
| Hispanic | 2.07 (1.39 – 3.09) | <0.001 |  | 2.56 (1.73 – 3.79) | <0.001 |
| Other/Unknown | 0.84 (0.59 – 1.20) | 0.342 |  | 0.92 (0.65 – 1.31) | 0.655 |
| Age |  |  |  |  |  |
| 18-34 | Ref |  |  | Ref |  |
| 35-44 | 1.23 (0.96 – 1.58) | 0.095 |  | 1.16 (0.91 – 1.48) | 0.230 |
| 45-54 | 0.92 (0.73 – 1.14) | 0.439 |  | 0.88 (0.71 – 1.10) | 0.252 |
| 55-64 | 0.88 (0.70 – 1.10) | 0.269 |  | 0.84 (0.67 – 1.05) | 0.128 |
| 65+ | 0.92 (0.68 – 1.25) | 0.593 |  | 0.89 (0.66 – 1.20) | 0.450 |
| Sex |  |  |  |  |  |
| Female | Ref |  |  | Ref |  |
| Male | 1.77 (1.11 – 2.83) | 0.016 |  | 1.79 (1.12 – 2.86) | 0.014 |
| Homeless |  |  |  |  |  |
| No | Ref |  |  | Ref |  |
| Yes | 3.73 (2.75 – 5.06) | <0.001 |  | 3.87 (2.85 – 5.24) | <0.001 |
| Rural/Urban |  |  |  |  |  |
| Metropolitan | Ref |  |  | Ref |  |
| Not Metropolitan | 0.74 (0.61 – 0.92) | 0.005 |  | 0.60 (0.49 – 0.73) | <0.001 |
| Index Year |  |  |  |  |  |
| 2007 – 2009 | Ref |  |  | Ref |  |
| 2010 – 2012 | 1.03 (0.87 – 1.23) | 0.737 |  | 1.02 (0.86 – 1.21) | 0.826 |
| 2013 – 2015 | 1.37 (1.14 – 1.65) | <0.001 |  | 1.39 (1.15 – 1.66) | <0.001 |
|  |  |  |  |  |  |
| West North Central | OR (95%CI) | P |  | OR (95%CI) | P |
| ADI |  |  |  |  |  |
| Q1 - Q3 | Ref |  |  | Ref |  |
| Q4 - Q5 | 1.04 (0.81 – 1.33) | 0.784 |  | 1.18 (0.93 – 1.51) | 0.181 |
| ISOL INDX |  |  |  |  |  |
| Q1 - Q3 | Ref |  |  |  |  |
| Q4 - Q5 | 1.99 (1.54 – 2.58) | <0.001 |  |  |  |
| Race/Ethnicity |  |  |  |  |  |
| NH White | Ref |  |  | Ref |  |
| NH Black | 4.09 (3.04 – 5.49) | <0.001 |  | 4.95 (3.70 – 6.60) | <0.001 |
| Hispanic | 1.06 (0.45 – 2.50) | 0.899 |  | 1.19 (0.51 – 2.81) | 0.688 |
| Other/Unknown | 0.62 (0.36 – 1.04) | 0.072 |  | 0.61 (0.36 – 1.04) | 0.068 |
| Age |  |  |  |  |  |
| 18-34 | Ref |  |  | Ref |  |
| 35-44 | 1.07 (0.72 – 1.59) | 0.738 |  | 1.06 (0.72 – 1.57) | 0.765 |
| 45-54 | 0.93 (0.66 – 1.32) | 0.689 |  | 0.91 (0.64 – 1.29) | 0.595 |
| 55-64 | 0.87 (0.60 – 1.25) | 0.438 |  | 0.84 (0.58 – 1.20) | 0.328 |
| 65+ | 1.21 (0.76 – 1.92) | 0.429 |  | 1.12 (0.71 – 1.78) | 0.628 |
| Sex |  |  |  |  |  |
| Female | Ref |  |  | Ref |  |
| Male | 2.94 (1.22 – 7.09) | 0.017 |  | 2.80 (1.16 – 6.77) | 0.022 |
| Homeless |  |  |  |  |  |
| No | Ref |  |  | Ref |  |
| Yes | 3.53 (2.16 – 5.77) | <0.001 |  | 3.90 (2.37 – 6.39) | <0.001 |
| Rural/Urban |  |  |  |  |  |
| Metropolitan | Ref |  |  | Ref |  |
| Not Metropolitan | 0.76 (0.58 – 0.99) | 0.046 |  | 0.63 (0.48 – 0.82) | <0.001 |
|  |  |  |  |  |  |
| Index Year |  |  |  |  |  |
| 2007 – 2009 | Ref |  |  | Ref |  |
| 2010 – 2012 | 1.21 (0.92 – 1.59) | 0.183 |  | 1.20 (0.91 – 1.58) | 0.202 |
| 2013 – 2015 | 1.25 (0.92 – 1.70) | 0.149 |  | 1.27 (0.94 – 1.73) | 0.119 |
|  |  |  |  |  |  |
| South Atlantic | OR (95%CI) | P |  | OR (95%CI) | P |
| ADI |  |  |  |  |  |
| Q1 - Q3 | Ref |  |  | Ref |  |
| Q4 - Q5 | 0.87 (0.80 – 0.95) | 0.001 |  | 1.01 (0.93 – 1.09) | 0.845 |
| ISOL INDX |  |  |  |  |  |
| Q1 - Q3 | Ref |  |  |  |  |
| Q4 - Q5 | 1.94 (1.78 – 2.12) | <0.001 |  |  |  |
| Race/Ethnicity |  |  |  |  |  |
| NH White | Ref |  |  | Ref |  |
| NH Black | 3.26 (2.98 – 3.57) | <0.001 |  | 4.22 (3.87 – 4.59) | <0.001 |
| Hispanic | 1.50 (1.21 – 1.85) | <0.001 |  | 1.81 (1.47 – 2.23) | <0.001 |
| Other/Unknown | 0.84 (0.71 – 1.01) | 0.058 |  | 0.93 (0.78 – 1.11) | 0.428 |
| Age |  |  |  |  |  |
| 18-34 | Ref |  |  | Ref |  |
| 35-44 | 0.88 (0.77 – 1.00) | 0.056 |  | 0.86 (0.76 – 0.98) | 0.026 |
| 45-54 | 0.79 (0.70 – 0.89) | <0.001 |  | 0.78 (0.69 – 0.87) | <0.001 |
| 55-64 | 0.98 (0.87 – 1.11) | 0.748 |  | 0.96 (0.85 – 1.08) | 0.469 |
| 65+ | 1.14 (0.95 – 1.37) | 0.157 |  | 1.10 (0.92 – 1.32) | 0.281 |
| Sex |  |  |  |  |  |
| Female | Ref |  |  | Ref |  |
| Male | 1.40 (1.18 – 1.67) | <0.001 |  | 1.41 (1.18 – 1.68) | <0.001 |
| Homeless |  |  |  |  |  |
| No | Ref |  |  | Ref |  |
| Yes | 4.91 (4.09 – 5.89) | <0.001 |  | 5.03 (4.20 – 6.03) | <0.001 |
| Rural/Urban |  |  |  |  |  |
| Metropolitan | Ref |  |  | Ref |  |
| Not Metropolitan | 0.79 (0.71 – 0.88) | <0.001 |  | 0.73 (0.66 – 0.81) | <0.001 |
|  |  |  |  |  |  |
| Index Year |  |  |  |  |  |
| 2007 – 2009 | Ref |  |  | Ref |  |
| 2010 – 2012 | 1.08 (0.98 – 1.20) | 0.107 |  | 1.06 (0.96 – 1.17) | 0.225 |
| 2013 – 2015 | 1.11 (1.00 – 1.23) | 0.054 |  | 1.09 (0.98 – 1.21) | 0.105 |
|  |  |  |  |  |  |
| East South Central | OR (95%CI) | P |  | OR (95%CI) | P |
| ADI |  |  |  |  |  |
| Q1 - Q3 | Ref |  |  | Ref |  |
| Q4 - Q5 | 1.27 (1.06 – 1.53) | 0.011 |  | 1.44 (1.20 – 1.73) | <0.001 |
| ISOL INDX |  |  |  |  |  |
| Q1 - Q3 | Ref |  |  |  |  |
| Q4 - Q5 | 1.96 (1.58 – 2.43) | <0.001 |  |  |  |
| Race/Ethnicity |  |  |  |  |  |
| NH White | Ref |  |  | Ref |  |
| NH Black | 3.01 (2.44 – 3.71) | <0.001 |  | 4.16 (3.46 – 5.01) | <0.001 |
| Hispanic | 1.87 (0.87 – 4.01) | 0.109 |  | 2.11 (0.99 – 4.49) | 0.053 |
| Other/Unknown | 0.82 (0.55 – 1.23) | 0.334 |  | 0.92 (0.62 – 1.36) | 0.660 |
| Age |  |  |  |  |  |
| 18-34 | Ref |  |  | Ref |  |
| 35-44 | 0.82 (0.62 – 1.10) | 0.182 |  | 0.80 (0.60 – 1.06) | 0.117 |
| 45-54 | 0.67 (0.51 – 0.86) | 0.002 |  | 0.66 (0.51 – 0.85) | 0.001 |
| 55-64 | 0.72 (0.55 – 0.94) | 0.016 |  | 0.71 (0.54 – 0.93) | 0.012 |
| 65+ | 0.76 (0.50 – 1.15) | 0.192 |  | 0.75 (0.49 – 1.14) | 0.173 |
| Sex |  |  |  |  |  |
| Female | Ref |  |  | Ref |  |
| Male | 0.95 (0.63 – 1.45) | 0.826 |  | 1.03 (0.68 – 1.56) | 0.901 |
| Homeless |  |  |  |  |  |
| No | Ref |  |  | Ref |  |
| Yes | 4.61 (3.03 – 7.00) | <0.001 |  | 4.89 (3.21 – 7.45) | <0.001 |
| Rural/Urban |  |  |  |  |  |
| Metropolitan | Ref |  |  | Ref |  |
| Not Metropolitan | 0.66 (0.54 – 0.80) | <0.001 |  | 0.62 (0.51 – 0.75) | <0.001 |
| Index Year |  |  |  |  |  |
| 2007 – 2009 | Ref |  |  | Ref |  |
| 2010 – 2012 | 0.80 (0.65 – 0.99) | 0.036 |  | 0.81 (0.66 – 1.00) | 0.050 |
| 2013 – 2015 | 1.06 (0.85 – 1.32) | 0.633 |  | 1.08 (0.87 – 1.35) | 0.479 |
|  |  |  |  |  |  |
| West South Central | OR (95%CI) | P |  | OR (95%CI) | P |
| ADI |  |  |  |  |  |
| Q1 - Q3 | Ref |  |  | Ref |  |
| Q4 - Q5 | 1.02 (0.90 – 1.16) | 0.732 |  | 1.19 (1.06 – 1.35) | 0.004 |
| ISOL INDX |  |  |  |  |  |
| Q1 - Q3 | Ref |  |  |  |  |
| Q4 - Q5 | 1.77 (1.55 – 2.03) | <0.001 |  |  |  |
| Race/Ethnicity |  |  |  |  |  |
| NH White | Ref |  |  | Ref |  |
| NH Black | 2.87 (2.50 – 3.30) | <0.001 |  | 3.52 (3.08 – 4.01) | <0.001 |
| Hispanic | 0.94 (0.77 – 1.15) | 0.566 |  | 1.16 (0.96 – 1.41) | 0.131 |
| Other/Unknown | 0.52 (0.40 – 0.68) | <0.001 |  | 0.58 (0.45 – 0.75) | <0.001 |
| Age |  |  |  |  |  |
| 18-34 | Ref |  |  | Ref |  |
| 35-44 | 0.88 (0.73 – 1.05) | 0.151 |  | 0.87 (0.73 – 1.04) | 0.115 |
| 45-54 | 0.70 (0.59 – 0.83) | <0.001 |  | 0.70 (0.59 – 0.83) | <0.001 |
| 55-64 | 0.79 (0.66 – 0.94) | 0.008 |  | 0.78 (0.66 – 0.93) | 0.006 |
| 65+ | 1.12 (0.86 – 1.46) | 0.398 |  | 1.11 (0.85 – 1.45) | 0.440 |
| Sex |  |  |  |  |  |
| Female | Ref |  |  | Ref |  |
| Male | 2.00 (1.44 – 2.78) | <0.001 |  | 2.02 (1.45 – 2.78) | <0.001 |
| Homeless |  |  |  |  |  |
| No | Ref |  |  | Ref |  |
| Yes | 6.09 (4.43 – 8.36) | <0.001 |  | 6.09 (4.44 – 8.35) | <0.001 |
| Rural/Urban |  |  |  |  |  |
| Metropolitan | Ref |  |  | Ref |  |
| Not Metropolitan | 0.65 (0.56 – 0.77) | <0.001 |  | 0.57 (0.49 – 0.67) | <0.001 |
| Index Year |  |  |  |  |  |
| 2007 – 2009 | Ref |  |  | Ref |  |
| 2010 – 2012 | 1.04 (0.90 – 1.20) | 0.615 |  | 1.03 (0.89 – 1.19) | 0.745 |
| 2013 – 2015 | 1.14 (0.97 – 1.33) | 0.102 |  | 1.15 (0.99 – 1.36) | 0.076 |
|  |  |  |  |  |  |
| Mountain | OR (95%CI) | P |  | OR (95%CI) | P |
| ADI |  |  |  |  |  |
| Q1 - Q3 | Ref |  |  | Ref |  |
| Q4 - Q5 | 0.84 (0.69 – 1.01) | 0.069 |  | 0.99 (0.83 – 1.19) | 0.955 |
| ISOL INDX |  |  |  |  |  |
| Q1 - Q3 | Ref |  |  |  |  |
| Q4 - Q5 | 1.74 (1.44 – 2.11) | <0.001 |  |  |  |
| Race/Ethnicity |  |  |  |  |  |
| NH White | Ref |  |  | Ref |  |
| NH Black | 2.80 (2.16 – 3.64) | <0.001 |  | 3.26 (2.52 – 4.21) | <0.001 |
| Hispanic | 1.13 (0.85 – 1.51) | 0.400 |  | 1.29 (0.97 – 1.70) | 0.081 |
| Other/Unknown | 0.66 (0.49 – 0.88) | 0.005 |  | 0.73 (0.54 – 0.97) | 0.028 |
| Age |  |  |  |  |  |
| 18-34 | Ref |  |  | Ref |  |
| 35-44 | 1.09 (0.83 – 1.44) | 0.536 |  | 1.05 (0.80 – 1.39) | 0.712 |
| 45-54 | 0.84 (0.65 – 1.09) | 0.184 |  | 0.84 (0.65 – 1.08) | 0.164 |
| 55-64 | 0.85 (0.65 – 1.11) | 0.234 |  | 0.84 (0.65 – 1.10) | 0.203 |
| 65+ | 1.05 (0.71 – 1.56) | 0.798 |  | 1.01 (0.68 – 1.49) | 0.977 |
| Sex |  |  |  |  |  |
| Female | Ref |  |  | Ref |  |
| Male | 2.14 (1.19 – 3.86) | 0.011 |  | 2.07 (1.15 – 3.73) | 0.015 |
| Homeless |  |  |  |  |  |
| No | Ref |  |  | Ref |  |
| Yes | 2.93 (2.05 – 4.18) | <0.001 |  | 3.07 (2.15 – 4.38) | <0.001 |
| Rural/Urban |  |  |  |  |  |
| Metropolitan | Ref |  |  | Ref |  |
| Not Metropolitan | 0.66 (0.52 – 0.83) | <0.001 |  | 0.61 (0.48 – 0.76) | <0.001 |
| Index Year |  |  |  |  |  |
| 2007 – 2009 | Ref |  |  | Ref |  |
| 2010 – 2012 | 1.09 (0.87 – 1.35) | 0.469 |  | 1.10 (0.88 – 1.37) | 0.395 |
| 2013 – 2015 | 0.97 (0.76 – 1.23) | 0.773 |  | 0.96 (0.76 – 1.22) | 0.759 |
|  |  |  |  |  |  |
| Pacific | OR (95%CI) | P |  | OR (95%CI) | P |
| ADI |  |  |  |  |  |
| Q1 - Q3 | Ref |  |  | Ref |  |
| Q4 - Q5 | 0.69 (0.61 – 0.78) | <0.001 |  | 0.70 (0.62 – 0.79) | <0.001 |
| ISOL INDX |  |  |  |  |  |
| Q1 - Q3 | Ref |  |  |  |  |
| Q4 - Q5 | 1.46 (1.30 – 1.64) | <0.001 |  |  |  |
| Race/Ethnicity |  |  |  |  |  |
| NH White | Ref |  |  | Ref |  |
| NH Black | 2.14 (1.83 – 2.49) | <0.001 |  | 2.43 (2.10 – 2.82) | <0.001 |
| Hispanic | 1.05 (0.88 – 1.26) | 0.595 |  | 1.20 (1.01 – 1.44) | 0.043 |
| Other/Unknown | 0.59 (0.49 – 0.71) | <0.001 |  | 0.64 (0.53 – 0.77) | <0.001 |
| Age |  |  |  |  |  |
| 18-34 | Ref |  |  | Ref |  |
| 35-44 | 0.97 (0.81 – 1.17) | 0.767 |  | 0.97 (0.81 – 1.17) | 0.767 |
| 45-54 | 0.83 (0.71 – 0.98) | 0.025 |  | 0.84 (0.71 – 0.98) | 0.031 |
| 55-64 | 0.90 (0.76 – 1.06) | 0.202 |  | 0.90 (0.76 – 1.06) | 0.191 |
| 65+ | 1.16 (0.92 – 1.46) | 0.202 |  | 1.15 (0.91 – 1.44) | 0.239 |
| Sex |  |  |  |  |  |
| Female | Ref |  |  | Ref |  |
| Male | 2.76 (1.81 – 4.22) | <0.001 |  | 2.73 (1.79 – 4.16) | <0.001 |
| Homeless |  |  |  |  |  |
| No | Ref |  |  | Ref |  |
| Yes | 3.67 (2.98 – 4.53) | <0.001 |  | 3.78 (3.06 – 4.65) | <0.001 |
| Rural/Urban |  |  |  |  |  |
| Metropolitan | Ref |  |  | Ref |  |
| Not Metropolitan | 0.60 (0.50 – 0.72) | <0.001 |  | 0.58 (0.48 – 0.69) | <0.001 |
| Index Year |  |  |  |  |  |
| 2007 – 2009 | Ref |  |  | Ref |  |
| 2010 – 2012 | 0.99 (0.86 – 1.14) | 0.878 |  | 0.99 (0.86 – 1.14) | 0.906 |
| 2013 – 2015 | 1.08 (0.92 – 1.25) | 0.375 |  | 1.07 (0.92 – 1.25) | 0.359 |
|  |  |  |  |  |  |

**eTable 6.** Multivariable Interaction term analysis between Area Deprivation Index (ADI) and Isolation Index (ISOL INDX) and HIV Incidence in U.S. Regions that Demonstrated a Significant ADI and ISOL INDX Interaction

|  | East South Central | |  | West South Central | |  | Pacific | |
| --- | --- | --- | --- | --- | --- | --- | --- | --- |
|  | aOR(95%CI)* | P |  | aOR(95%CI)* | P |  | aOR(95%CI)* | P |
| ADI-ISOL INDX |  |  |  |  |  |  |  |  |
| LwADI-LwISO (High SES-Low Segregation) | Ref |  |  | Ref |  |  | Ref |  |
| LwADI-HgISOL | 1.63 (1.27 – 2.11) | <0.001 |  | 1.45 (1.23 – 1.71) | <0.001 |  | 1.20 (1.05 – 1.39) | 0.010 |
| HgADI-LwISOL | 0.88 (0.63 – 1.25) | 0.474 |  | 0.71 (0.57 – 0.88) | 0.002 |  | 0.51 (0.42 – 0.60) | <0.001 |
| HgADI-HgISOL (Low SES-High Segregation) | 2.44 (1.90 – 3.13) | <0.001 |  | 1.83 (1.57 – 2.14) | <0.001 |  | 1.12 (0.95 – 1.32) | 0.195 |
| Race/Ethnicity |  |  |  |  |  |  |  |  |
| NH White | Ref |  |  | Ref |  |  | Ref |  |
| NH Black | 2.91 (2.35 – 3.59) | <0.001 |  | 2.83 (2.46 – 3.26) | <0.001 |  | 2.13 (1.82 – 2.48) | <0.001 |
| Hispanic | 1.83 (0.85 – 3.92) | 0.122 |  | 0.92 (0.76 – 1.13) | 0.440 |  | 1.03 (0.86 – 1.24) | 0.724 |
| Other/Unknown | 0.81 (0.54 – 1.21) | 0.310 |  | 0.52 (0.40 – 0.67) | <0.001 |  | 0.59 (0.49 – 0.71) | <0.001 |
| Homeless |  |  |  |  |  |  |  |  |
| No | Ref |  |  | Ref |  |  | Ref |  |
| Yes | 4.67 (3.07 – 7.11) | <0.001 |  | 6.02 (4.38 – 8.28) | <0.001 |  | 3.71 (3.01 – 4.58) | <0.001 |
| Rural/Urban |  |  |  |  |  |  |  |  |
| Metropolitan | Ref |  |  | Ref |  |  | Ref |  |
| Not Metropolitan | 067 (0.55 – 0.82) | <0.001 |  | 0.69 (0.58 – 0.82) | <0.001 |  | 0.63 (0.52 – 0.75) | <0.001 |

aOR: Adjusted Odds Ratio; P: p-value; ADI: Area Deprivation Index; ISOL INDX: Isolation Index;

*Mutually adjusted odds ratio. Age, sex and index year are not included since they are matching variables.

**East South Central** Alabama, Kentucky, Mississippi, Tennessee

**West South Central** Arkansas, Louisiana, Oklahoma, Texas

**Pacific** California, Oregon, Washington
